# The Ability to Induce Microtubule Acetylation Is a General Feature of Formin Proteins

**DOI:** 10.1371/journal.pone.0048041

**Published:** 2012-10-24

**Authors:** Susan F. Thurston, Wojciech A. Kulacz, Sahir Shaikh, Jonathan M. Lee, John W. Copeland

**Affiliations:** 1 Department of Cellular and Molecular Medicine, Faculty of Medicine, University of Ottawa, Ottawa, Ontario, Canada; 2 Department of Biochemistry, Microbiology and Immunology, Faculty of Medicine, University of Ottawa, Ottawa, Ontario, Canada; Georgia Health Sciences University, United States of America

## Abstract

Cytoplasmic microtubules exist as distinct dynamic and stable populations within the cell. Stable microtubules direct and maintain cell polarity and it is thought that their stabilization is dependent on coordinative organization between the microtubule network and the actin cytoskeleton. A growing body of work suggests that some members of the formin family of actin remodeling proteins also regulate microtubule organization and stability. For example, we showed previously that expression of the novel formin INF1 is sufficient to induce microtubule stabilization and tubulin acetylation, but not tubulin detyrosination. An important issue with respect to the relationship between formins and microtubules is the determination of which formin domains mediate microtubule stabilization. INF1 has a distinct microtubule-binding domain at its C-terminus and the endogenous INF1 protein is associated with the microtubule network. Surprisingly, the INF1 microtubule-binding domain is not essential for INF1-induced microtubule acetylation. We show here that expression of the isolated FH1 + FH2 functional unit of INF1 is sufficient to induce microtubule acetylation independent of the INF1 microtubule-binding domain. It is not yet clear whether or not microtubule stabilization is a general property of all mammalian formins; therefore we expressed constitutively active derivatives of thirteen of the fifteen mammalian formin proteins in HeLa and NIH3T3 cells and measured their effects on stress fiber formation, MT organization and MT acetylation. We found that expression of the FH1 + FH2 unit of the majority of mammalian formins is sufficient to induce microtubule acetylation. Our results suggest that the regulation of microtubule acetylation is likely a general formin activity and that the FH2 should be thought of as a dual-function domain capable of regulating both actin and microtubule networks.

## Introduction

The correct establishment of cell polarity is essential for a number of critical cellular events, including asymmetric cell division, cell migration, specialization of cellular function and tissue formation. In addition, defective cell polarity is associated with the progression and metastasis of a variety of cancers [1], [2], [3], [4], [5]. In most contexts, cell polarity is controlled by the coordinated actions of the actin cytoskeleton and microtubule (MT) network [6], [7], [8], [9], [10]. During the establishment of polarity, an asymmetric stable MT network is generated which serves as a directional marker within the cell. This process is thought to rely on “bridging factors” which link MTs to actin filaments to guide formation of a stable MT network. One candidate for this activity is the formin family of cytoskeletal remodelling proteins [8], [11].

Formins are highly conserved throughout evolution with multiple family members found in all eukaryotes. Formin proteins are distinguished by the presence of two regions of homology, Formin Homology 1 (FH1) and Formin Homology 2 (FH2), and these comprise the primary cytoskeletal regulatory unit. These proteins have been best characterized for their role in regulating actin dynamics through FH1–FH2, but a growing body of work suggests that they are also key regulators of MT organization and stability [12]. For example, Formin2-induced actin assembly is required for normal positioning of the spindle during oogenesis [13], [14], while its Drosophila homologue, *cappuccino*, is required for normal MT function during fly oocyte maturation [15], [16]. Over-expression of active derivatives of the formins Dia1 and FHOD1 induce stress fiber formation and the concomitant co-alignment of the MT network [17], [18]. Beyond intracellular organization of MTs, the formins Dia1, Dia2, Dia3 and INF1 have also been shown to promote MT stability. During mitosis, Dia3 is recruited to the kinetochore where it promotes microtubule stabilization and kinetochore-microtubule attachment [19]. Over-expression of Dia1 or Dia2 induces MT stabilization and detyrosination [20]. These effects are likely mediated both by a direct interaction of these proteins with MTs as well as with other MT-associated proteins [20], [21], [22], [23]. Similarly, expression of the novel formin INF1 also induces MT stabilization and acetylation, but, unlike Dia1 and Dia2, INF1 does not induce tubulin detyrosination [24].

The mechanism by which formins regulate MT dynamics *in vivo* is still unknown. Of the fifteen mammalian formins, six have now been shown to bind MTs directly (Dia1, Dia2, Fmn1, Fmn2, INF1 and INF2) [23], [24], [25], [26], [27]. Dia1, Dia2, formin2 and INF2 bind MTs directly through their FH2 domain *in vitro* [23], [26], [27]. A recent analysis showed that Dia1, Dia2 and INF2 bind MTs with a high affinity (60–95 nM) and, although each of these proteins interacts with MTs through the same domain, the mode of interaction must vary [26]. Dia1 and INF2 bind tubulin with an approximate 3∶1 tubulin to formin ratio while Dia2 binds tubulin with a 1∶1 ratio. Formins may also use other domains separate from FH2 to interact with the MT network. Formin1 (Fmn1) binds MTs through a large domain N-terminal to FH1 and FH2 [25]. We have shown that INF1 binds MTs directly through a distinct bipartite MT-binding domain (MTBD) at the C-terminus [24]. INF1 is also unique among formins in that the endogenous protein is found specifically associated with MTs; this is not the case for the other five formins shown to bind MTs. In characterizing the INF1 MTBD we showed that over-expression of full-length INF1 was sufficient to induce stress fiber formation, co-alignment of the MT network, MT stabilization and MT acetylation, but not detyrosination. MT acetylation could also be induced by expression of the isolated INF1 MTBD. Surprisingly, expression of an INF1 derivative lacking the MTBD was also sufficient to induce MT acetylation. This derivative contained FH1 and FH2, as well as other conserved regions, but unlike Dia1 and Dia2, expression of the isolated INF1 FH2 domain was not sufficient to induce MT acetylation [24].

In this study, we perform a deletional analysis of INF1 to determine which regions of the protein are required to induce MT acetylation independent of the MTBD. Through this analysis we show that this activity resides in the FH1–FH2 unit of INF1. Given that the ability to regulate MT acetylation resides in the FH1–FH2 unit and that other formins also bind MTs through this region we sought to determine if expression of the FH1−FH2 of other formins is sufficient to induce MT acetylation. We show that eleven of the thirteen mammalian formins tested are able to induce MT acetylation and that this ability is likely a general feature of formin proteins.

## Results

**Figure 1:**
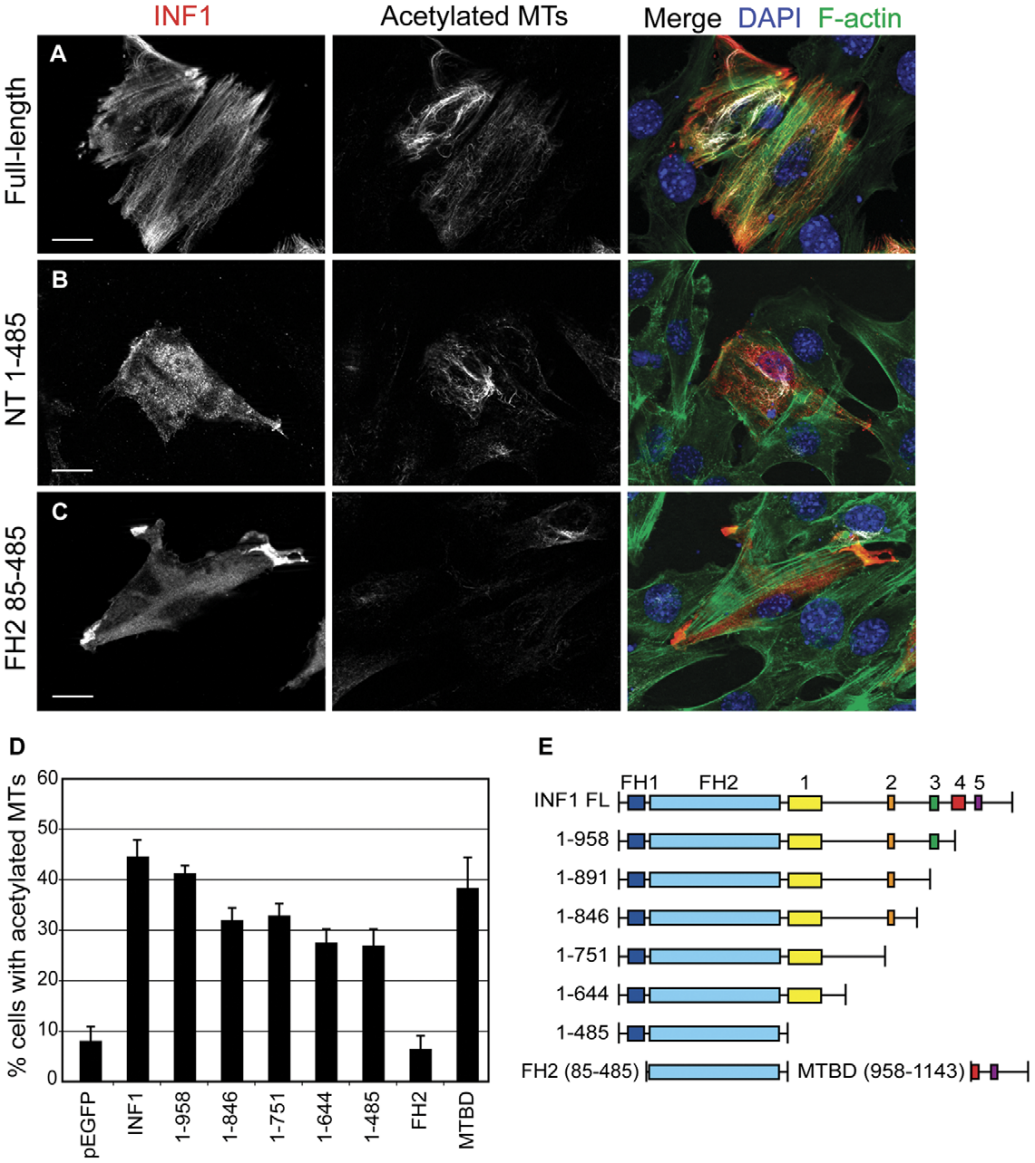
The FH1/FH2 unit of INF1, but not FH2 alone is sufficient to induce microtubule acetylation. Over-expressed INF1 derivatives were visualized with an N-terminal myc epitope tag (left panels). Acetylated microtubules were detected using anti acetyl-alpha-tubulin antibody (middle panels). F-actin was detected with fluorescein phalloidin (green, merged image, right panels) **A**) Expression of full-length INF1 by transient transfection induces MT acetylation in NIH 3T3 fibroblasts. **B**) Expression of the INF1 N-terminus (AA 1–485), containing the FH1 and FH2 domain module, is also able to efficiently induce MT acetylation. **C**) Expression of the INF1 FH2 domain does not induce MT acetylation above background. **D**) Quantification of the effects of expression of INF1 deletion derivatives on MT acetylation in transiently transfected cells. Full-length INF1, INF1 1–958 and the INF1 MTBD (microtubule Binding Domain) induce MT acetylation to a similar extent (40–45%). Expression of the 1–846, 1–751, 1–644 and 1–485 also induce MT acetylation to a reduced but significant extent (27–30%). Expression of the INF1 FH2 domain does not induce MT acetylation above the background of empty pEGFP-C1 vector alone. N = 5 with greater than 100 cells counted for each derivative. Error bars represent standard error of the mean (SEM). **E**) Schematic of INF1 derivatives used. FH1-Formin Homology 1, FH2-Formin Homology 2, Numbers 1–5 represent conserved domains FHDC1-5 as in [24], [45].

We previously showed that over-expression of INF1 was sufficient to induce MT stabilization and acetylation and that expression of the INF1 MTBD was sufficient for this effect. Surprisingly, however, expression of an INF1 derivative lacking the MTBD also induced MT acetylation. Unlike the case with mDia1 and mDia2 [23], expression of the INF1 FH2 domain was not sufficient to induce MT stabilization or acetylation [24]. To further our understanding of how INF1 regulates MT acetylation and stabilization, we sought to identify which domains of INF1 are required to induce MT acetylation independent of the MTBD. We generated a series of deletion derivatives that progressively removed the conserved motifs found in the INF1 C-terminus (Figure 1). These deletions were expressed in NIH 3T3 fibroblasts by transient transfection and the effects on microtubule acetylation were determined by immunofluorescence using an anti-acetylated α-tubulin antibody. As previously observed, approximately 50% of the cells expressing full-length INF1 were enriched for acetylated MTs (Figure 1A, D), while only 5% of control cells expressing mCherry displayed the same phenotype (Figure 1D). To our surprise, deletion of the entire region C-terminal to the INF1 FH2 did not have a significant effect on the ability of INF1 to induce MT acetylation (INF1.1–485, Figure 1B, D). Nor did deletion of the conserved region N-terminal to the FH1 motif (INF1.25–485, Figure 1D). However, deletion of the FH1 motif, leaving the FH2 domain in isolation, eliminated INF1-induced MT acetylation, as observed previously [24]. Thus, the FH1–FH2 domains of INF1 comprise a minimal functional domain capable of inducing MT acetylation and none of the conserved C-terminal motifs of INF1 is necessary for this activity.

The observation that the conserved FH1–FH2 functional unit is both necessary and sufficient to induce MT acetylation prompted us to consider if this activity might be a general feature of other formin proteins. To answer this question, we generated a series of deletion derivatives consisting of the isolated FH1–FH2 domains of DAAM1&2, Dia1, 2&3, FHOD1&3, Fmn1, FMNL1, 2&3, and INF2. To aid in the standardization of the comparison, each of these derivatives were sub-cloned into the same expression vector encoding an N-terminal myc epitope tag. Thus, we were able to survey the activities of an additional twelve of the fifteen vertebrate formins.

**Figure 2:**
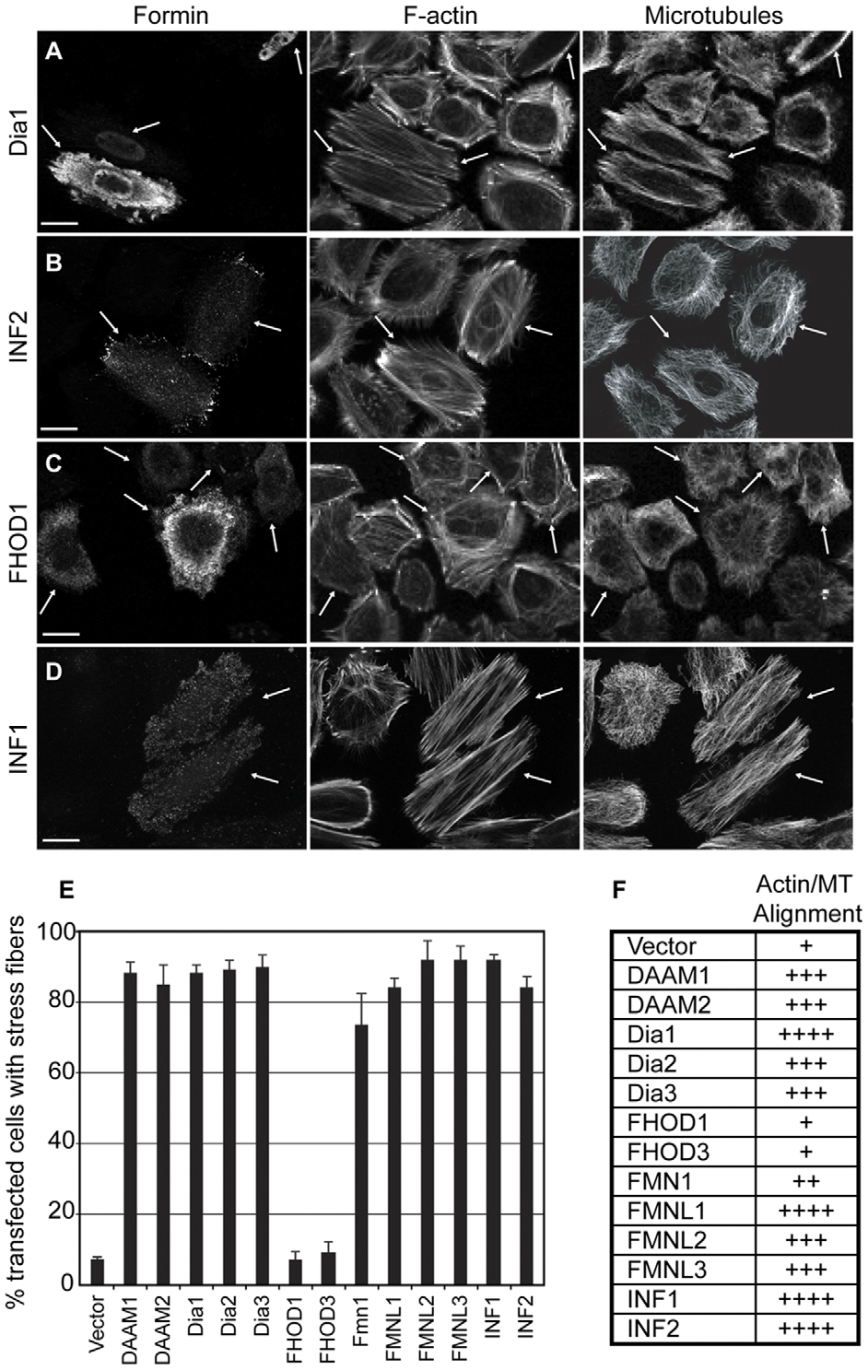
Induction of F-actin and microtubule alignment by expression of the formin FH1–FH2 module. FH1–FH2 containing derivatives of thirteen of the fifteen mammalian formins were over-expressed by transient transfection in HeLa cells and the effects on cellular morphology were observed by immunofluorescence. **A**) Over-expression of Dia1 FH1–FH2 (left panel) induces stress fiber formation (middle panel) and their concomitant co-alignment with the microtubule network (right panel). **B**) INF2 FH1–FH2 expression also induces co-alignment of stress fibers and microtubules. **C**) Over-expression of FHOD1 FH1–FH2 fails to induce stress fiber formation. **D**) INF1 FH1–FH2 expression induces stress fiber formation and microtubule co-alignment. **E**) Quantification of stress fiber formation. The number of transfected cells with obvious increased stress fiber formation was counted. N = 3, with greater than 100 cells counted per sample. Error bars represent SEM. **F**) Summary of stress-fiber alignment with the microtubule network as assessed visually by immunofluorescence.

We first compared the relative performance of these proteins in a known FH1–FH2 dependent activity. Over-expression of constitutively active FH1–FH2 containing derivatives of either Dia1, FHOD1 or INF1 has been shown to induce stress fiber formation and concomitant co-alignment of the actin filaments with the microtubule network [17],[18],[24]. This effect is most easily observed in HeLa cells [17] and less so in fibroblasts [18]. Thus we chose to examine the effects of formin over-expression on longitudinal stress fiber formation and co-alignment of actin filaments and MTs in HeLa cells. Myc-tagged derivatives of each of the tested proteins were expressed by transient transfection and the effects on cellular morphology were determined by immunofluorescence (Figures 2 & S1). Expression of FH1FH2 derivatives of DAAM1, DAAM2, Dia1, Dia2, Dia3, Fmn1, FMNL1, FMNL2, FMNL3, INF1, and INF2 induced stress fiber formation, cellular elongation and MT alignment. The only exceptions being the FHOD1 and FHOD3 FH1FH2 derivatives which had essentially no effect on stress fiber formation, MT organization or cellular morphology (Figures 2C & S1).

**Figure 3:**
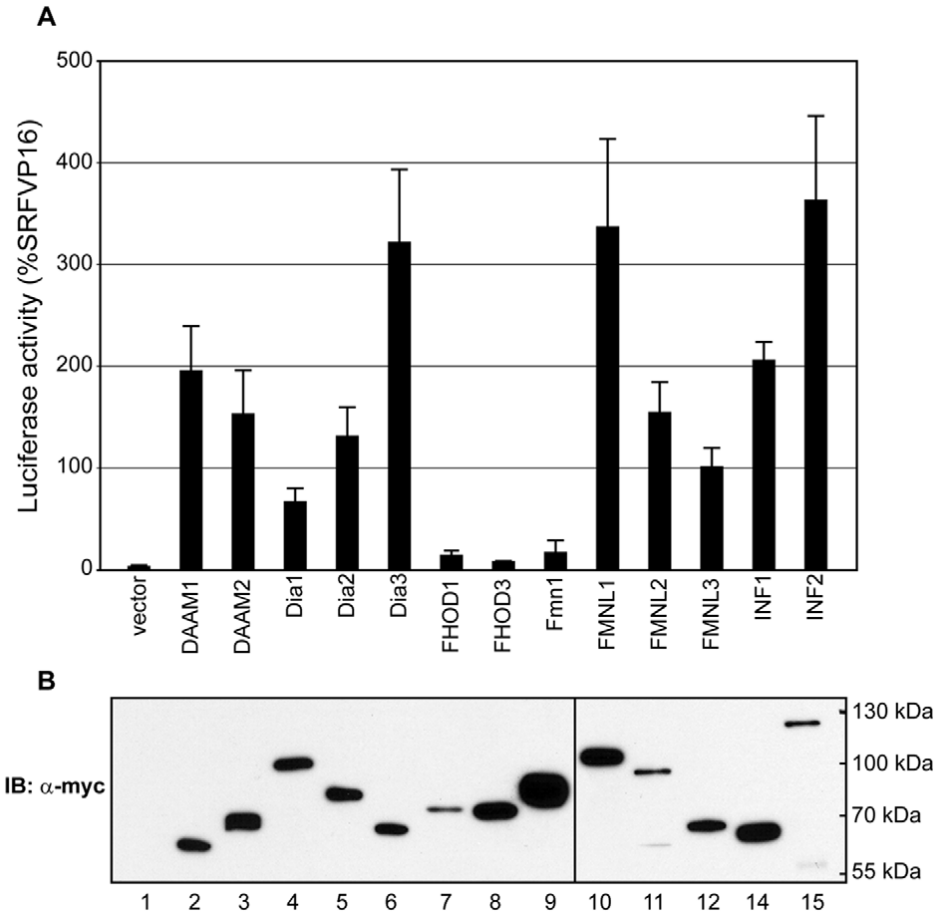
Expression of formin FH1–FH2 derivatives that induce stress fiber formation also induce activation of an SRF reporter gene. **A**) NIH 3T3 fibroblasts were transiently transfected with an SRF luciferase reporter gene and the indicated formin FH1FH2 derivative. Formin derivatives that induce stress fiber formation also induce activation of the SRF reporter gene. Expression of FHOD1, FHOD3 and Fmn1 FH1FH2 derivatives fail to induce robust activation of the SRF reporter consistent with their effects on stress fiber formation. Reporter activation is expressed relative to an SRF-VP16 control fusion protein. N = 4, error bars  =  SEM. **B**) Equivalent samples of transfected cell lysates from (A) were subjected to SDS-PAGE and immunoblotted to detect the myc epitope tag on the expressed formin proteins; lane order matches the order in (A).

To extend our analysis, we next compared the ability of each of the formin FH1FH2 derivatives to activate an SRF reporter gene that responds to changes in actin dynamics. This assay measures depletion of cellular G-actin stores and is independent of the type of F-actin structure formed [28]. It therefore provides an objective, quantitative method to compare effects on actin dynamics. We found that between formins there was a dramatic variation in their ability to induce activation of the SRF reporter gene in a transient transfection assay. As reported previously [24], [28], [29], [30], expression of mDia1, mDia2, INF1, FMNL2 and FMNL3 all induced potent activation of the reporter (Figure 3A). We also found that expression of INF2, FMNL1, DAAM1, DAAM2 and Dia3 similarly resulted in potent levels of activation. As with the immunofluorescence analysis we found that the FH1FH2 derivatives of FHOD1 and FHOD3 had very little effect on activation of the reporter gene (Figure 3A), similar to what we had observed for Fmn1 in an earlier study (Copeland 2004). As expected, there was a strong correlation between the ability to induce SRF activation and the formation of actin stress fibers. Some variation in levels of expression could be detected by immunoblotting for the myc epitope tag (Figure 3B), but this did not correlate with the relative level of activity in this assay and could not account for the variation in activity observed between each formin derivative (Figure 3B, compare lanes 11 and 15 to lanes 7–9).

**Figure 4:**
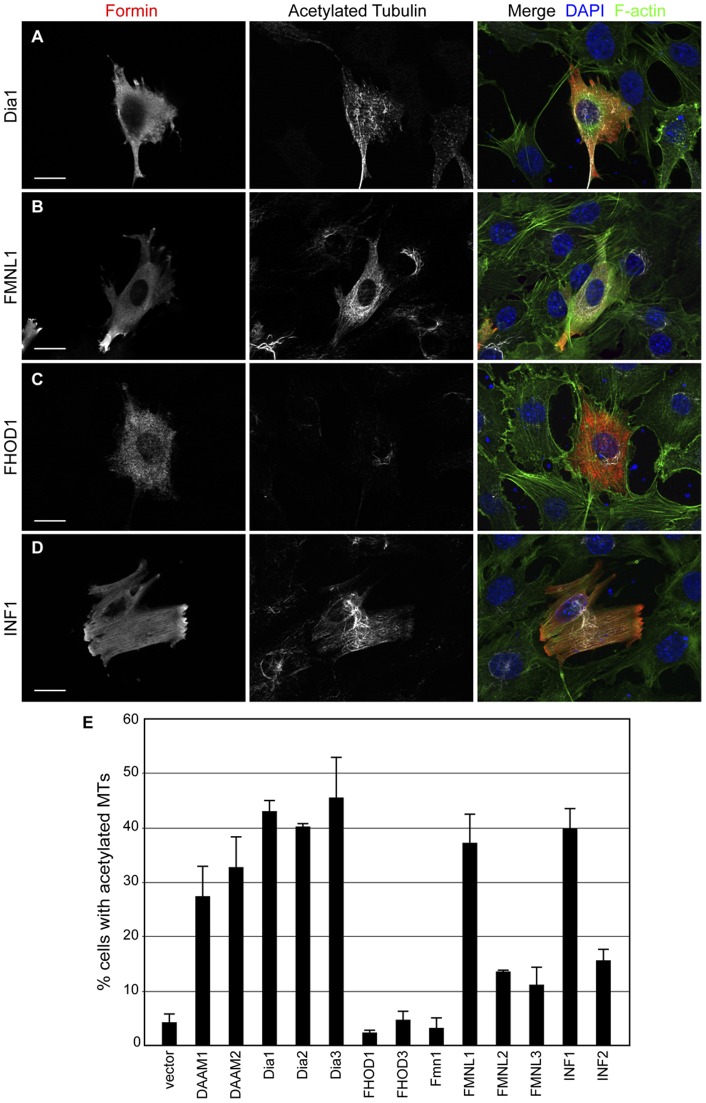
Microtubule acetylation induced by expression of formin FH1FH2 derivatives. FH1–FH2 containing formin derivatives were expressed in NIH 3T3 fibroblasts by transient transfection. Their effects on microtubule acetylation were assessed by immunofluorescence. **A**) Expression of Dia 1 (red, left panel) induces microtubule acetylation (white, middle panel). The F-actin network is also shown in the merged image (green, right panel). **B**) Expression of FMNL1 (red, right panel) induces microtubule acetylation (white, middle panel). **C**) Expression of the FHOD1 FH1–FH2 derivative (red, left panel) fails to induce microtubule acetylation (white, middle panel). **D**) Expression of INF1 (red, left panel) induces microtubule acetylation (white, middle panel). **E**) Quantification of effects of formin FH1FH2 derivative expression on microtubule acetylation. The percent of transfected cells with increased microtubule acetylation was determined by immunofluorescence. N = 5, with greater than 100 cells counted per sample. Error bars represent SEM.

Having compared the relative effectiveness of the various formins in their ability to induce stress fiber formation and their concomitant co-alignment with the microtubule network, we then sought to compare the relative ability of each to affect microtubule acetylation. As above, each of the FH1–FH2 formin derivatives were expressed by transient transfection in NIH 3T3 cells and the effect on microtubule acetylation visualized by immunofluorescence (Figure 4). Expression of mCherry alone served as a negative control while expression of INF1, Dia1 and Dia2 served as positive benchmarks. We found that expression of INF1, Dia1 or Dia2 all induced microtubule acetylation to a similar extent, i.e. in approximately 50% of transfected cells. As might be expected, similar results were obtained with the Diaphanous-Related Formins Dia3, DAAM1 and DAAM2 as these three proteins are highly homologous to Dia1. Similarly, we found that expression of FMNL1 was also able to induce microtubule acetylation with comparable efficiency. However, despite their similarity to FMNL1, expression of either FMNL2 or FMNL3 was much less efficient at inducing MT acetylation, with approximately 15% of transfected cells displaying acetylated microtubules. Similar results were obtained with INF2 (Figure 4). Finally, as with the stress fiber formation and SRF reporter gene assays, we found that expression of FH1FH2-containing derivatives of either Fmn1, FHOD1 or FHOD3 had no effect on microtubule acetylation over background (approximately 5% of transfected cells). Thus the FH1–FH2 dependent activity of the formins fall into three groups: strong acetylators (Dia1,2,&3, DAAM1&2, FMNL1, and INF1), weak acetylators (FMNL2&3 and INF2) and non-acetylators (FHOD1,3, and Fmn1).

**Figure 5:**
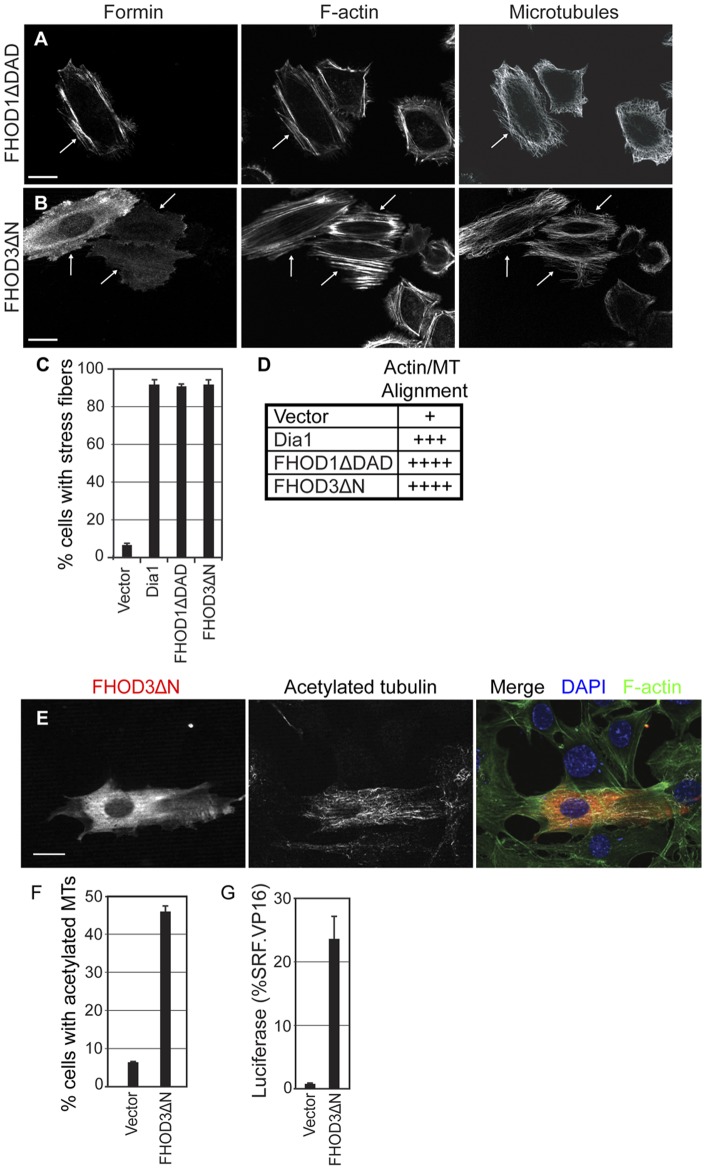
Larger derivatives of FHOD1 and FHOD3 induce stress fiber formation and co-alignment with microtubules. **A, B**) FHOD1ΔDAD and FHOD 3ΔN were expressed in HeLa cells by transient transfection. Expression of either formin derivative (left panel) induced stress fiber formation (middle panel) and concomitant co-alignment with microtubules (right panel). **C**) The number of transfected cells with increased stress fiber formation was determined by immunofluorescence. N = 3, >100 cells per sample, error bars represent SEM. **D**) Summary of stress fiber and microtubule co-alignment. Dia1 FH1FH2 served as a positive control. **E**) **FHOD3ΔN expression induces microtubule acetylation.** FHOD3ΔN expression (red, left panel) induces microtubule acetylation (white, middle panel) in NIH 3T3 fibroblasts. F-actin is also shown in the merged image (green, right panel). We were unable to obtain significant levels of expression of FHOD1ΔDAD in NIH 3T3 cells. **F**) Quantification of effects of FHOD3ΔN expression on microtubule acetylation. The percent of transfected cells with increased microtubule acetylation was determined by immunofluorescence. N = 5, >100 cells per sample. Error bars represent SEM. **G**) Expression of FHOD3ΔN induces activation of an SRF reporter gene in NIH 3T3 fibroblasts (see Figure 3). N = 3, error bars represent SEM.

In all of the assays described above we were struck by the low activity of the FH1–FH2 containing derivatives of FHOD1 and FHOD3. This result was somewhat unexpected given previous reports showing FHOD1 expression induces stress fiber formation [18], [31]. In these previous studies, however, FHOD1 was rendered constitutively active by deletion of the DAD and it has been proposed that a domain within the FHOD1 N-terminus is required for regulation of actin dynamics by this protein [32], [33], [34]. Thus we investigated the ability of larger FHOD1 and FHOD3 derivatives to affect actin and MT dynamics in our assays (Figure 5). Expression of FHOD1ΔDAD or FHOD3ΔN in HeLa cells induced stress fiber formation and MT co-alignment (Figure 5A–D) similar to other formins and as had been previously observed for FHOD1 [18]. As these derivatives were able to induce stress fiber formation and MT co-alignment in HeLa cells we next wanted to assay their effects on MT acetylation in NIH 3T3 cells. We were unable to obtain significant levels of expression of FHOD1ΔDAD and therefore unable to acquire reliable data with this derivative in this assay. The FHOD3ΔN derivative, however, was well expressed in these cells and its expression was sufficient to induce MT acetylation to a level similar to strong acetylators such as Dia1 (Figure 5E, F). Consistent with its ability to induce stress fiber formation in HeLa cells, we also found that FHOD3ΔN expression was sufficient to induce activation of the actin-responsive SRF reporter gene (Figure 5G). Therefore, in comparison to the FH1–FH2 derivative, FHOD3ΔN has gained the ability to both regulate actin dynamics and induce MT acetylation.

## Discussion

We showed previously that INF1 possesses a MTBD whose expression is sufficient to induce MT acetylation. Surprisingly, expression of an INF1 derivative lacking the MTBD also induces acetylation [24]. Our results show that this second, MTBD-independent, acetylation activity resides in the FH1–FH2 functional unit of INF1. This would imply that INF1 regulates MT acetylation and stabilization through two independent pathways, one MTBD-dependent and one FH1–FH2 dependent. The FH1 dependency suggests that this is likely acting through a mechanism distinct from that observed for the FH2-dependent induction of MT acetylation by Dia1 and Dia2. It also raised the possibility that the ability to induce MT acetylation might be a general feature of formin proteins. Here we show that expression of the FH1–FH2 domains of 10 of the 13 vertebrate formins is sufficient to induce MT acetylation. Some formins, however, are more effective at this activity than others. Expression of DAAM1, DAAM2, Dia1, Dia2, Dia3, FMNL1 and INF1 FH1FH2 derivatives induced MT acetylation in 30–50% of transfected cells. We classify these as “strong” acetylators. FMNL2, FMNL3 and INF2 FH1–FH2 expression consistently induced MT acetylation in roughly 15% of transfected cells and are classified as “weak” acetylators. Last FH1–FH2 derivatives of FHOD1, FHOD3 and Fmn1 are non-acetylators which fail to induce acetylation above background.

What may account for the difference between formins in their ability to induce MT acetylation? The mechanism behind formin-induced MT acetylation remains to be determined and it is a strong possibility that there are multiple independent pathways mediating this effect. There are conflicting reports as to the effects of formins on the activity of the tubulin deacetylase HDAC6. Dia1 is proposed to inhibit HDAC6 activity [35] while Dia2 is proposed to activate it [36]. It has also been suggested that Dia1 stabilizes MTs in part through its association with the MT-binding protein EB1. This interaction is proposed to be mediated by a YEDXR peptide motif in the Dia1 FH2 domain [21]. This sequence, while present in Dia1,2&3, is not found in the other “strong” acetylators, DAAM1&2, FMNL1, INF1. Nor is there conservation of the 3 lysine residues in FH2 that have been proposed to mediate formin-dependent regulation of microtubule organization [17]. Indeed, we observed that all formins able to induce stress fiber formation also induced co-alignment of the MT network (Figures 2 & S1). We also find no obvious correlation between the ability to induce MT acetylation and the ability to bind MTs directly; none of the tested FH1–FH2 derivatives show an obvious association with MTs when over-expressed in either HeLa or NIH 3T3 cells (Figures 2,3, S1 & S2). Similarly, the Dia1, Dia2 and INF2 FH2 domains have all recently been shown to be able to bind MTs directly [26], but INF2 is not a strong acetylator while Dia1 and Dia2 are (Figure 4E). This is consistent with our observation that INF1-induced MT acetylation is FH1-dependent and therefore not likely to be solely contingent on a FH2-MT interaction (Figure 1) [24]. The recent identification of the tubulin acetyl transferase [37] will allow an informed investigation into how formins interact with this part of the MT modification machinery.

### MT acetylation and actin dynamics

The relationship between the ability of formins to induce MT acetylation and to affect actin dynamics is not clear. Previous work has suggested that the ability to regulate actin dynamics is not required for Dia2-induced MT stabilization [23]. Consistent with this, we do not see any direct relationship between the effects of a given formin on MT acetylation and its relative effects on actin dynamics. For example, FMNL1, Dia1 and Dia2 are all strong inducers of MT acetylation (Figure 4), but only Dia1 and Dia2 are strong nucleators of actin polymerization *in vitro*, while FMNL1 is considered a weak nucleator [38]. Similarly, we do not see any absolute relationship between the ability to induce activation of an actin sensitive SRF reporter gene and the ability to induce MT acetylation (compare Figures 3 and 4E). In this case, Dia3, FMNL1 and INF2 are all potent activators of this reporter, but INF2 is a weak inducer of MT acetylation. Nonetheless, our results do suggest that there may be some association between the ability to regulate actin dynamics and MT acetylation and this connection is supported by earlier work showing Dia1-induced MT stabilization is inhibited by disruption of actin filaments [35]. Consistent with this, we find that FH1–FH2 derivatives of Fmn1, FHOD1 and FHOD3 are poor inducers of stress fiber formation and SRF activation and these derivatives also fail to induce MT acetylation above background. Further evidence for a potential link comes from our experiments with FHOD3. The FH1–FH2 derivative of FHOD3 (AA 776–1401) has only a very weak effect in our assays, while the larger FHOD3ΔN (AA 219–1401) derivative is notably stronger in stress fiber formation, SRF activation and MT acetylation (Figures 2, 3, 4 & 5). If MT acetylation is independent of the effects of FH2 on actin dynamics, then why does the larger derivative of FHOD3 gain both activities? This raises intriguing questions as to how FH2 activity is modified by additional domains in the protein and how the effects on actin and MT dynamics are related, if at all.

In summary we show that expression of the FH1–FH2 functional unit of the majority of mammalian formins is sufficient to induce MT acetylation. The results obtained with a larger derivative of FHOD3 suggest the possibility that larger derivatives of FHOD1 and Fmn1 may also likely regulate MT acetylation [25]. If so, then this would suggest that formins are multi-functional proteins able to regulate both actin and MT dynamics. We found previously that INF1 expression induces MT acetylation, but not detyrosination, and for this reason we restricted ourselves to only monitoring acetylation in this study. Other formins (such as DAAM1, Dia1 and Dia2), however, are able to induce both; it is not known if formins affect other MT modifications such as poly-glutamylation and poly-glycylation. The varied array of MT post-translational modifications is proposed to generate distinct MT networks each with a specific role in intracellular organization and function [39]. The ability of formins to regulate multiple MT modifications, in coordination with their effects on actin dynamics, suggests that this family of proteins may represent a central node for the coordinated regulation of cytoskeletal dynamics. Further investigation will be required to establish how formins interact with distinct components of the MT regulatory machinery to mediate their effects.

## Materials and Methods

### Cell culture

NIH 3T3 fibroblasts were a gift from Dr Richard Treisman and cultured in DMEM +10% FBS in 10% CO_2_ as previously described [40]. HeLa cells were a gift from Dr Laura Trinkle-Mulcahy and were grown in DMEM +10%FBS in 5% CO_2_ as previously described [17].

### Plasmids

Dia1,2, Fmn1, and FMNL2,3 deletion derivatives were previously described [28], [29], [30]. The hDia2 cDNA (designated here as Dia3) was obtained from Dr S. Gasman [41], Daam1&2 cDNAs were obtained from Dr T. Yamaguchi [42], FMNL1 and INF2 cDNAs were obtained from Dr H. Higgs [43], [44], FHOD3 cDNA (KIAA1695) was obtained from the Kazusa cDNA project. FH1–FH2 deletion constructs were generated by PCR to generate fragments containing the indicated codons: DAAM1 (NP_080378)  =  codons 527–1042; DAAM2 (NP_001008232.2)  =  codons 517–1080; Dia1 (NP_031884.1)  =  codons 567–1255; Dia2 (NP_062644.1)  =  codons 532–1170; Dia3 (NP_009293.1)  =  codons 548–1097; FHOD1 (NP_037373.2)  =  codons 569–1164; FHOD3 (EAX01381)  =  codons 776–1401; Fmn1 (AAI38037.1)  =  codons 771–1334; FMNL1 (NP_062653.2)  = 449–1091; FMNL2 (NP_443137.2) = 504–1094; FMNL3 (NP_783863.4) = 491–1028; INF1 (NP_203751.2) = 1–485; INF2 (NP_940803.2) = 419–1272. The PCR fragments were subcloned into pEFNBRSS or pEF.linkTAG [28] using standard techniques. Both of these vectors drive expression using the EF1α promoter and encode an N-terminal myc epitope tag. The presence of an identical tag on all constructs allowed for direct comparison of expression levels by immunoblotting.

### Transfection

NIH 3T3 cells were plated at a density of 125,000 cells/well in 6-well plates. Cells were transfected the next day using PEI as in [30]. Briefly, 1.5 µg total plasmid DNA was diluted in 50 µL Optimem, 5 µL of 1 mg/mL PEI was added and the mixture was incubated for 25–30 minutes at room temperature. The DNA/PEI mix was added to cells in 1 mL of Optimem and left for 5 hours under normal culture conditions. At the end of 5 hours the media was replaced with 2 mL of the appropriate culture medium. Similar procedures were performed for transfection of HeLa cells. For immunofluorescence in NIH 3T3 cells 0.3 µg of formin expression plasmid was used for each sample, 0.5 µg was used for HeLa cells. The SRF reporter gene assays were performed as in [40]. Briefly, 50 ng of the SRF reporter p3D. ALuc and 0.25 µg of the transfection control reporter pMLV-LacZ was used for each sample. 50 ng of pEF-SRF.VP16 was included as a positive control in each reporter gene experiment.

### Immunofluorescence

Cells were prepared for immunofluorescence as in [24]. Briefly, cells cultured on acid-washed glass coverslips were fixed for 10 minutes directly in 2 mL of 4% para-formaldehyde freshly prepared in 1xPBS. Following fixation the cells were permeabilized for 20 minutes in 0.3% Triton-X-100, 5% Donor Bovine Serum (DBS) in 1xPBS. The coverslips were washed in 1xPBS and incubated with the appropriate primary antibody in 0.03% Triton-X-100, 5% DBS in 1xPBS for 1 hour at room temperature. The coverslips were washed 3 times in 1xPBS and then incubated with secondary antibody in the same solution for 1 hour at room temperature. After washing in 1xPBS the coverslips were mounted in Vectashield with DAPI and sealed with nail polish. Primary antibodies used were: Rabbit anti-myc, 1∶200 dilution (Santa Cruz Biotech); mouse anti-acetylated α-tubulin, 1∶1000 (Sigma). Secondary antibodies were: Alexa594 donkey anti-rabbit, 1∶200 (Invitrogen); Cy5 Donkey anti-mouse, 1∶200 (Jackson Labs). F-actin was detected with fluorescein phalloidin, 1∶200 (Invitrogen).

### MT acetylation assays

NIH 3T3 fibroblasts were plated on 6-well plates at 175,000 cells/well and transfected as above. Following transfection, the cells were cultured in starvation media (DMEM +0.5% FCS) and incubated for 48 hours. The cells were prepared for immunofluorescence as above. Acetylated MTs were detected with mouse anti acetyl-α-tubulin and Cy5 anti-mouse secondary. F-actin was detected with fluorescein phalloidin and the over-expressed formin was detected using Rabbit anti-myc as above. To prevent bias in visual quantification of MT acetylation, images of the first 100 transfected cells for each sample were captured “blind” without visualizing the acetylated MT signal in far-red. The images were again scored “blind” after capture with the identity of the over-expressed formin kept concealed until completion of each experiment. Results were confirmed by a second observer for at least one independent replicate of each formin construct.

## Supporting Information

Figure S1 **Effects of formin FH1–FH2 derivative expression on stress fiber formation and microtubule organization in HeLa cells.** As in Figure 2, FH1–FH2 containing derivatives of the indicated formins were expressed by transient transfection in HeLa cells. Formin expression (left panel,) was detected by immunofluorescence by virtue of an encoded N-terminal myc epitope tag. F-actin (middle panel) was detected with phalloidin and microtubules were detected with an anti α-tubulin antibody (right panel).
(TIF)

Figure S2 **Effects of formin FH1–FH2 derivative expression on microtubule acetylation in fibroblasts.** As in Figure 4, FH1–FH2 containing derivatives of the indicated formins were expressed by transient transfection in NIH 3T3 cells. Formin expression (left panel, red in merged image) was detected by immunofluorescence by virtue of the encoded N-terminal myc epitope tag. Acetylated microtubules were detected with an anti-acetylated α-tubulin antibody (middle panel, white in merged image). F-actin was detected with Alexa488-phalloidin (green, left panel).
(TIF)
