# Supplementary figures and images for: The Ability to Induce Microtubule Acetylation Is a General Feature of Formin Proteins
Source: PLoS One. 2012 Oct 24;7(10):e48041. doi: 10.1371/journal.pone.0048041 (PMC3480493; doi:10.1371/journal.pone.0048041)

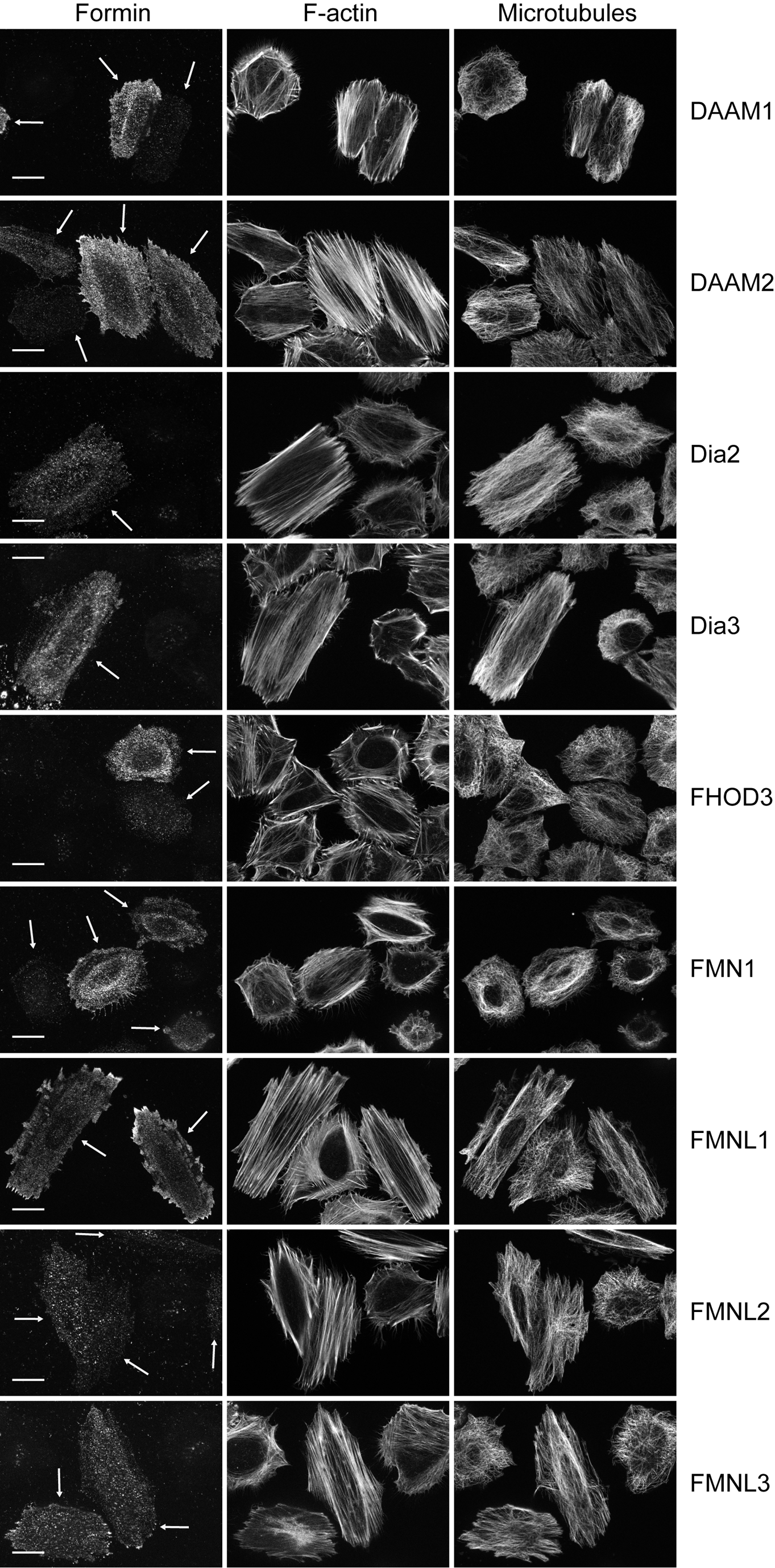

Supplement: Figure S1 — Effects of formin FH1–FH2 derivative expression on stress fiber formation and microtubule organization in HeLa cells. As in Figure 2, FH1–FH2 containing derivatives of the indicated formins were expressed by transient transfection in HeLa cells. Formin expression (left panel,) was detected by immunofluorescence by virtue of an encoded N-terminal myc epitope tag. F-actin (middle panel) was detected with phalloidin and microtubules were detected with an anti α-tubulin antibody (right panel). (TIF) [file pone.0048041.s001.tif]

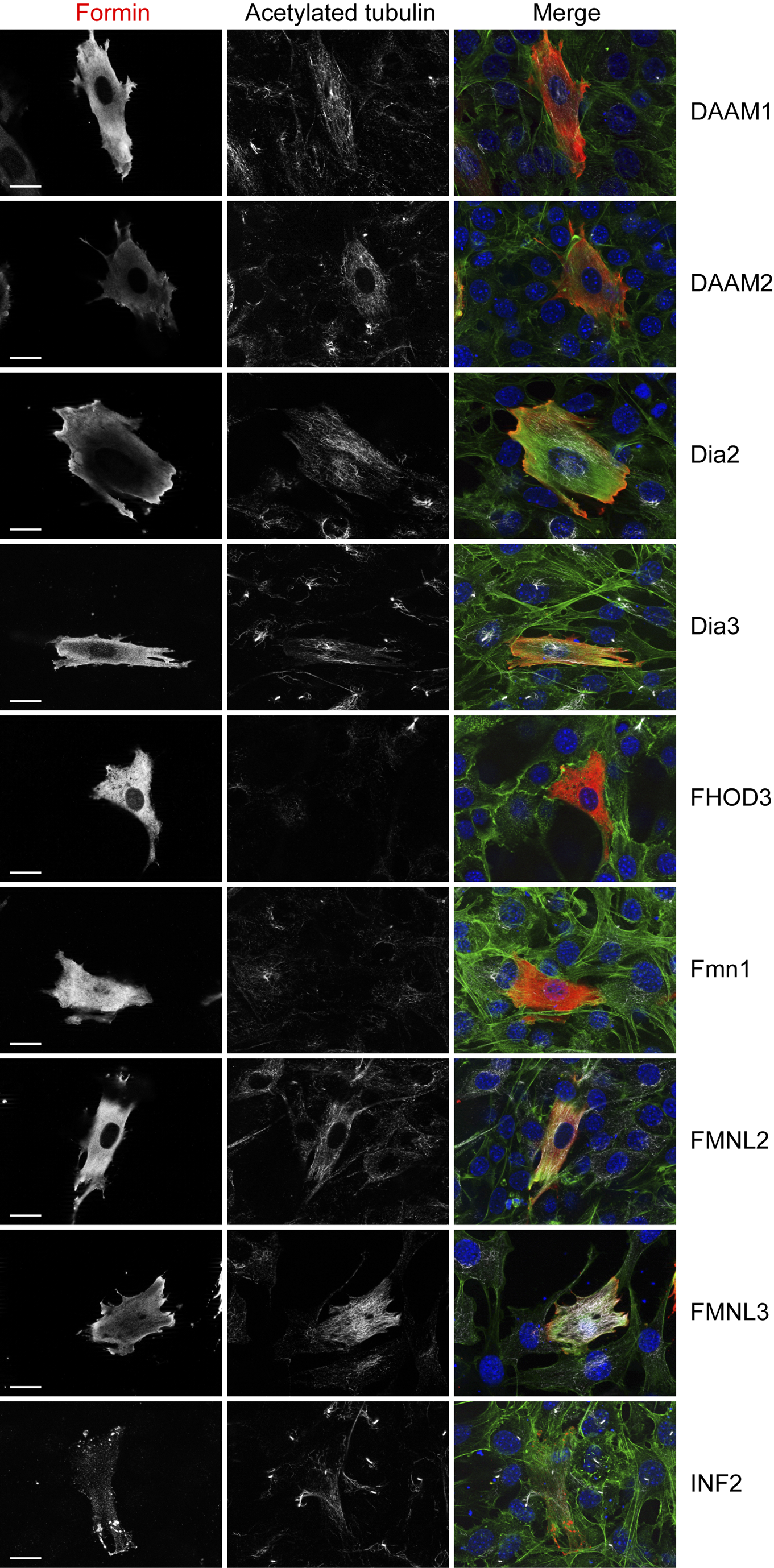

Supplement: Figure S2 — Effects of formin FH1–FH2 derivative expression on microtubule acetylation in fibroblasts. As in Figure 4, FH1–FH2 containing derivatives of the indicated formins were expressed by transient transfection in NIH 3T3 cells. Formin expression (left panel, red in merged image) was detected by immunofluorescence by virtue of the encoded N-terminal myc epitope tag. Acetylated microtubules were detected with an anti-acetylated α-tubulin antibody (middle panel, white in merged image). F-actin was detected with Alexa488-phalloidin (green, left panel). (TIF) [file pone.0048041.s002.tif]
